# Technology for the prevention of antimicrobial resistance and healthcare-associated infections; 2017 Geneva IPC-Think Tank (Part 2)

**DOI:** 10.1186/s13756-019-0538-y

**Published:** 2019-05-22

**Authors:** Walter Zingg, Benjamin J. Park, Julie Storr, Raheelah Ahmad, Carolyn Tarrant, Enrique Castro-Sanchez, Eli Perencevich, Andreas Widmer, Karl-Heinz Krause, Claire Kilpatrick, Sara Tomczyk, Benedetta Allegranzi, Denise Cardo, Didier Pittet, the 2017 Geneva IPC-Think Tank, Mohamed Abbas, Raheelah Ahmad, Benedetta Allegranzi, Antoine Andremont, Mike Bell, Michael Borg, Denise Cardo, Yehuda Carmeli, Enrique Castro-Sanchez, John Conly, Philippe Eggimann, Petra Gastmeier, M. Lindsay Grayson, Stephan Harbarth, Marcela Hernandez, Loreen Herwaldt, Alison Holmes, John A. Jernigan, Claire Kilpatrick, Amy Kolwaite, Karl-Heinz Krause, Elaine Larson, Sarah Masson-Roy, Shaheen Mehtar, Marc Mendelson, Ling Moi Lin, Andreea Moldovan, Dominique Monnet, Babacar Ndoye, Peter Nthumba, Folasade Ogunsola, Ben Park, Eli Perencevich, Didier Pittet, Matthew Samore, Wing Hong Seto, Arjun Srinivasan, Julie Storr, Evelina Tacconelli, Carolyn Tarrant, Maha Talaat, Sara Tomczyk, Maria Virginia Villegas, Andreas Voss, Tim Walsh, Andreas Widmer, Walter Zingg

**Affiliations:** 1 Infection control programme and WHO collaborating center, University of Geneva Hospitals and Faculty of Medicine, 4 Rue Gabrielle Perret-Gentil, 1211, Geneva 14, Switzerland; 2 US Centers for Disease Control and Prevention, Atlanta, GA USA; 3 Infection Prevention and Control Global Unit, World Health Organization, Geneva, Switzerland; 4 National Institute for Health Research in Healthcare Associated Infection and Antimicrobial Resistance, Imperial College London, London, UK; 5 Department of Health Sciences, University of Leicester, Leicester, UK; 6 Carver College of Medicine, University of Iowa, Iowa City, USA; 7 Infection Control Programme, University Hospitals of Basel, Basel, Switzerland; 8 Institute of Genetics and Genomics, University of Geneva, Geneva, Switzerland

**Keywords:** Technology, Infection prevention and control, Microbiome, Whole genome sequencing, Copper, Hand hygiene, CDC, ECDC, WHO

## Abstract

**Background:**

The high burden of healthcare-associated infections (HAIs) and antimicrobial resistance (AMR) is partially due to excessive antimicrobial use both in human and animal medicine worldwide. How can technology help to overcome challenges in infection prevention and control (IPC) and to prevent HAI and emerging AMR?

**Methods:**

In June 2017, 42 international experts convened in Geneva, Switzerland to discuss four potential domains of technology in IPC and AMR: 1) role and potential contribution of microbiome research; 2) whole genome sequencing; 3) effectiveness and benefit of antimicrobial environmental surfaces; and 4) future research in hand hygiene.

**Results:**

Research on the microbiome could expand understanding of antimicrobial use and also the role of probiotics or even faecal transplantation for therapeutic purposes. Whole genome sequencing will provide new insights in modes of transmission of infectious diseases. Although it is a powerful tool for public health epidemiology, some challenges with interpretation and costs still need to be addressed. The effectiveness and cost-effectiveness of antimicrobially coated or treated environmental high-touch surfaces requires further research before they can be recommended for routine use. Hand hygiene implementation can be advanced, where technological enhancement of surveillance, technique and compliance are coupled with reminders for healthcare professionals.

**Conclusions:**

The four domains of technological innovation contribute to the prevention of HAI and AMR at different levels. Microbiome research may offer innovative concepts for future prevention, whole genome sequencing could detect new modes of transmission and become an additional tool for effective public health epidemiology, antimicrobial surfaces might help to decrease the environment as source of transmission but continue to raise more questions than answers, and technological innovation may have a role in improving surveillance approaches and supporting best practice in hand hygiene.

## Background

A panel of 42 international experts with backgrounds in infection prevention and control (IPC), microbiology, infectious diseases, public health, psychology, medical technology, and social sciences, convened for two days at the Geneva Think Tank on IPC and antimicrobial resistance (AMR) in June 2017 under the auspices of the US Centers for Disease Control and Prevention (CDC), the World Health Organization (WHO), and the University of Geneva Hospitals and Faculty of Medicine (HUG). The experts were from both high- and low-and-middle-income countries, and from all five continents. The objectives were to develop a vision on IPC and AMR and to agree on a road map for research and public health activities. Three dimensions on IPC and AMR were discussed: 1) implementation of IPC and antimicrobial stewardship; 2) technology in IPC and AMR; and 3) broadening the global IPC network. This is the second in a series of three 2017 Geneva IPC-Think Tank papers; it summarises the discussions about technology in the prevention of healthcare-associated infections (HAIs) and AMR.

## Methods

Four domains of technological innovation for IPC and AMR were assessed: 1) role and potential of microbiome research; 2) potential of whole genome sequencing (WGS); 3) effectiveness and cost-effectiveness of antimicrobial surfaces; and 4) future research in hand hygiene surveillance and improvement. The domains were selected as a consensus by CDC, WHO, and HUG. Each domain was introduced with a short summary presentation by one of the experts, before they were allocated to four focus groups. The discussions were guided by a moderator, tape recorded, and documented by writers. The experts were invited to express their personal views, expectations and concerns in relation to using technology in IPC and AMR. At the end of the four discussion rounds a plenary session was organised, where abstracts from the groups were summarized.

## Results

### The role of the microbiome

The populations of microbial species living on or in the human body are known as the microbiota; the microbiome is the total of the genes of the microbiota. While the total number of human cells and microbial cells is similar, there are 50 times more bacterial genes than human genes in or on a human individual (10^6^ versus 2 × 10^4^) [1]. The majority of the microbiota, and of the microbiome, is found in the intestines. Research on the microbiome as part of our immune system is rapidly progressing but has not yet emerged into a mature technology in relation to IPC or AMR.

The use of antibiotics interferes with the balance of the microbiota, which results in diseases such as *Clostridoides difficile* infection (CDI), or can promote the selection of multidrug-resistance microorganisms (MDROs) [2]. Improved understanding on the impact of antimicrobials on the microbiome could improve stewardship efforts. Probiotics and faecal transplantation are two examples of microbiota restoration -related interventions with potential for prevention and treatment [3–5], respectively. Probiotics can be preventive (e.g. in the prevention of necrotizing enterocolitis in preterm infants) but also have the potential to be harmful (e.g. in causing probiotic species-related infection in immunocompromised patients). IPC professionals may be concerned particularly with the latter, but microbiota-based intervention as a prevention strategy for AMR is innovative with beneficial potential. Microbiome research can help in directing researchers to ask the right questions on benefits and risks of such interventions, and to design clinical studies with new products. Faecal transplantation is a different way of influencing the microbiota, and has been successfully tested to treat recurrent CDI. However, in terms of AMR, additional research is still needed to understand the impact and best uses of this strategy.

### Whole genome sequencing

Whole genome sequencing is another rapidly growing field of research within IPC and AMR, and reports using this technology are growing in numbers. Organisations such as the US CDC and the European Centre for Disease Prevention and Control (ECDC) promote the use of WGS because of its many strengths such as detailed species identification, detection of antimicrobial resistance at the mechanism level, reproducibility, and objective comparison of data. For example, in 2018, all Swiss university hospitals are routinely using this technology to identify clusters and epidemics on a local, national and international scale. Combining information on species and antimicrobial resistance using WGS would be a particularly interesting advance in the monitoring of AMR on regional, national and international levels.

This technology is still growing and a number of challenges need to be addressed. At least today, phenotypic information is needed to inform genotypic interpretation, and data interpretation will constantly change over time. Although some countries are investing in national database platforms, there is a lack of valid international reference platforms. Today, WGS still is a research tool – although advancing rapidly – and as such, many researchers use their own standards and references for data interpretation. Epidemiological use of WGS on the other hand would need valid standards and international libraries, even if the latter grow and change over time.

Costs are currently still high, but new, less expensive kits are becoming available. However, for low-and-middle-income countries, these kits might still be too expensive. Whole genome sequencing needs highly trained people, particularly for data analysis and interpretation, which adds to the costs of this technology. Open source software makes interpretation easier and faster than in the past, and software integrating epidemiology data with those from WGS are getting on the market.

### Antimicrobial surfaces

There is agreement that hospital cleaning is key for patient safety and that materials and design should facilitate cleanliness. Surfaces in hospital environments are often contaminated with microorganisms, however, to what extent this contamination contributes to MDRO transmission is not yet clear [6, 7]. It is known that cleaning practices are inconsistent, and that surfaces re-contaminate rapidly after cleaning.

Antimicrobial surfaces are intended to keep bacterial loads low, and thus reduce the risk of pathogen transmission. The added value of antimicrobial surfaces to a clean hospital environment is largely unknown, and other technologies may contribute more to patient safety. Ideally, antimicrobial surfaces would not need maintenance, but exhibit permanent effectiveness. However, such a technology is not yet in sight. Copper appears to be effective in reducing bioburden, but is expensive and may even be stolen from hospitals (there is an illegal market for copper in some countries). Low-and-middle-income countries do not have the resources to buy copper or other even more sophisticated surfaces. Furthermore, all marketed products today need to be manually cleaned. Thus, cost-effectiveness has been difficult to be demonstrated. A number of questions (rather than answers) were raised for antimicrobial surfaces by the experts: which surfaces in a hospital should be coated: high-touch surfaces (used by different people) such as door handles, surfaces in the patient zone including textiles, toilets and showers, or any surface in a patient room? Could there be resistance to surface antimicrobials? Maybe, surfaces and equipment that are carefully designed for easy cleaning would be more effective and cost effective in the end.

### Hand hygiene

Hand hygiene is the most widely promoted single intervention for the prevention of HAI and transmission of MDROs. Direct hand hygiene observation is considered the best method to assess compliance, but, among key ongoing challenges with hand hygiene improvement, the Hawthorne effect limits its validity [8]. There may be better methods of measurement and feedback than direct hand hygiene observation: there has been an explosion of efforts to incorporate technology into hand hygiene measurement. For example, a study using video surveillance did highlight some success [9], while surveillance using handrub counters showed effectiveness only after implementing a positive deviance strategy [10]. Non-alcohol-based hand cleansing agents (with sporicidal activity) were considered to improve compliance and act longer (particularly relevant for surgery), but no such product is available for daily routine use at the frontline.

The experts perceived automated surveillance positively. They decided that more research should be invested there, with a view to providing more credible data than is currently obtained in many settings. The use of new un-obtrusive surveillance technologies would be preferable, even at the cost of accuracy. However, is such technology affordable? Compared to the time needed for direct hand hygiene observation, automated surveillance might be cost-effective and would give IPC professionals time to do other tasks. An emerging question is how new technological innovation could guide healthcare workers to perform hand hygiene at the right moment. Colour changing products or electronic reminders were proposed as imaginable, forward-looking strategies. The ideal hand hygiene agent would have to be fast acting, long-lasting, quick-drying, and skin-friendly with a wide range of effectiveness (also towards spores and non-enveloped viruses). Longer-lasting agents would be good, but more important than duration is effectiveness. In addition to effectiveness, other aspects such as price, local production, and ecological properties (waste) must be considered for future products. Appropriate exposure time, application technique and optimal number and placement of handrub dispensers are additional research questions.

## Discussion

The IPC community has focussed on best practices for the prevention of HAI and AMR in recent years, including aspects related to implementation and behaviour change. However, at the same time new technologies are being evaluated or are becoming more broadly available. The microbiota has a number of functions in nutrition, metabolism, and maturation of the immune system, and thus, research on this topic going beyond probiotics and faecal transplantation, including diagnostics, should draw the attention of the IPC community. The benefits in the prevention of HAI and AMR need to be more fully established. Whole genome sequencing is a promising technology and a powerful tool for future epidemiology in both IPC and AMR. Public health interests are different from research interests, and using WGS for public health purposes requires coordinated standards and infrastructure. International organisations should work together with national public health bodies, experts and researchers of the IPC community to foster outputs of interest for public health such as quality assurance of WGS, standardised methodologies, and international libraries. Antimicrobial surfaces are unlikely to play an important role in the short term given their expense and lack of efficacy data supporting wide adoption. There is agreement that the evidence for measurable effectiveness is poor and thus, broad recommendations of antimicrobial surfaces cannot be justified at this time. Hand hygiene research now needs to be taken to the next level; in particular, accurate surveillance, appropriate technique, required amount of handrub, and automatic reminders for healthcare professionals would be of interest. However, given that hand hygiene is and remains driven by the behaviour of healthcare professionals, all future aspects taking into account new technology should be considered within a multimodal improvement strategy.

The time available to discuss technology was short, limiting the aim to obtain consensus on a road map for all four topics. The role of the microbiota in IPC was a new concept, and except from using probiotics and applying faecal transplantation to treat CDI and to address AMR, there was limited time to discuss this concept in a wider range (e.g. the role of the hospital microbiota). In the discussion on WGS, some experts expressed concerns about this technology being already sufficiently advanced and reproducible to serve as a gold standard. Thus, both microbiota and WGS will be the two topics to be discussed in the next Geneva think tank.

## Conclusion

The four domains of technological innovation contribute to the prevention of HAI and AMR at different levels. Microbiome research may offer innovative concepts for future prevention, whole genome sequencing could detect new modes of transmission and become an additional tool for effective public health epidemiology, antimicrobial surfaces might help to decrease the environment as source of transmission but continue to raise more questions than answers, and technological innovation may have a role in improving surveillance approaches and supporting best practice in hand hygiene.

## Abbreviations

AMR: Antimicrobial resistance
ARHAI: Antimicrobial Resistance and Healthcare-Associated Infection (−network)
CDC: US Centers for Disease Control and Prevention
CDI: *Clostridioides difficile* infection
ECDC: European Centre for Disease Prevention and Control
HAI: Healthcare-associated infection
HUG: University of Geneva Hospitals
IPC: Infection prevention and control
MDRO: Multidrug-resistant organism
WGS: Whole genome sequencing
WHO: World Health Organization
